# Professional boundary blurring and role dilution: a taxonomy of diabetes educator activities in Malaysian primary care

**DOI:** 10.1186/s12875-026-03489-x

**Published:** 2026-07-29

**Authors:** Zalina Libasin, Nur Jihan Noris, Pangie Bakit, Nik Dewi Delina Nik Mohd Kamil, Intan Syafinaz Saimy

**Affiliations:** 1 Institut Kesihatan Negara, Shah Alam, Selangor Malaysia; 2 Universiti Malaysia Sabah, Kota Kinabalu, Sabah Malaysia

**Keywords:** Diabetes educators, Taxonomy, Primary health clinics, Professional boundaries, Role dilution, Health services management

## Abstract

**Background:**

This study develops a comprehensive taxonomy of Diabetes Educators’ (DEs) activities in Malaysian primary health clinics (PHCs) to analyze the “blurring” of professional boundaries between specialized education and general clinical duties.

**Methods:**

A sequential mixed-methods design was used. Phase 1 defined role expectations through document analysis and expert interviews. Phase 2 validated activities through five-day direct observations of 51 DEs across 43 clinics. Each trained observer monitored the same DE for five consecutive working days. All DE activities, including clinical duties, administrative tasks, and other related activities (discussions, meetings, community outreach), were recorded. Findings were based on frequency rather than time-weighted workload.

**Results:**

The taxonomy identified 146 activities across five domains. While 56.85% were patient-centric, results revealed significant role dilution, with 32.19% of activities dedicated to non-diabetes care. Activities were further classified into core specialized, routine clinical, and clerical/delegable categories, revealing significant systemic task-shifting. ‘Clerical/delegable’ (39.04%) highlighted a heavy clerical burden. This research captured the empirical operational reality of DEs, providing evidence of systemic task-shifting in high-volume settings.

**Conclusions:**

The taxonomy clarifies the role of DEs by delineating “core specialized” from “clerical/delegable” tasks. This framework provides an essential evidence-based tool for Family Medicine Specialists to optimize the distribution of human resources, mitigate role dilution, and improve the quality of care in high-volume PHC settings.

**Supplementary Information:**

The online version contains supplementary material available at https://doi.org/10.1186/s12875-026-03489-x.

## Background

Diabetes is a global epidemic, affecting over 830 million people worldwide, with significant prevalence in low- and middle-income countries [1]. In Malaysia, diabetes is a major non-communicable disease (NCD), nearly 15.6% of Malaysian adults had diabetes in 2023, and a high percentage remain undiagnosed [2]. Diabetes substantially increases the risk of cardiovascular disease, a leading cause of death in the country [3].

Additionally, effective diabetes management requires an interdisciplinary approach that integrates medical treatment, patient education, and self-management support. In Malaysia, almost 80% of patients seek treatment at public healthcare facilities, primarily primary health clinics (PHC) [4, 5]. The Ministry of Health (MOH) publishes guidelines to standardize care [6]. Central to this care are Diabetes Educators (DEs), who play an integral role in patient education, promoting adherence, guiding lifestyle changes, and fostering self-management skills [6, 7]. Despite their importance, DEs face significant challenges, including high workloads, competing responsibilities (like screening counter duties), and resource limitations in PHCs [5, 8].

Furthermore, the complexity of diabetes management highlights the need for a more structured definition of DE roles. Globally, established frameworks such as the Australian Diabetes Educators Association (ADEA) scope of practice and the American Association of Diabetes Care & Education Specialists (ADCES) competencies offer robust guidance on professional capabilities and ideal clinical standards [9, 10]. However, these international benchmarks predominantly outline high-level competencies in well-resourced environments rather than capturing granular, day-to-day operational activities. While existing Malaysian guidelines and standard operating procedures outline the general roles of DEs in managing diabetes care, they do not specify or map out their comprehensive daily tasks and workflows [6, 11]. This leaves a critical empirical gap regarding how theoretical scopes of practice translate into actual workplace execution within public healthcare systems.

Developing a comprehensive taxonomy of DEs’ activities in PHCs is necessary for several reasons. Firstly, in an inherently interdisciplinary setting, a clear framework of DEs’ specific roles and responsibilities is vital for clarifying contributions and facilitating better teamwork among physicians, nurses, and dietitians [12]. Secondly, such a taxonomy enhances the quality of care by providing a structured approach to evaluate DEs’ performance and allowing PHCs with limited resources to prioritize high-impact activities (e.g., patient education over administrative tasks). Finally, a clear taxonomy helps standardize care delivery across different PHCs, reducing variability and ensuring all patients receive a consistent, high standard of education [13].

Accordingly, this study aims to establish a comprehensive taxonomy of DEs’ activities at PHCs to serve as a framework for delineating roles, prioritizing tasks, and fostering collaboration among healthcare professionals. Grounded in professional boundary theory and task-shifting models, this taxonomy moves beyond a simple list of activities to serve as a conceptual tool for analyzing the ‘blurring’ of boundaries between specialized diabetes education and general clinical duties. Such a framing allows for a critical interrogation of how systemic factors, including staffing shortages and under-digitalization, influence the daily operational reality of the DE role. Specifically, task-shifting models describe the strategic redistribution or delegation of clinical duties within health workforce teams, moving specific interventions from highly trained healthcare personnel to workers with targeted training to enhance health system efficiency and equity [14].

In high-volume primary care settings, however, this structural redistribution can trigger complex socio-professional negotiations described by professional boundary theory. Rather than remaining fixed, occupational jurisdictions are continuously contested through daily practice as either ‘boundary making’ or ‘boundary blurring’. Boundary making is a process of demarcation in which a professional group actively protects its exclusive ownership of specialized tasks to preserve occupational status and intellectual jurisdiction. Conversely, boundary blurring involves a cooperative process of hybridization in which overlapping roles cause strict professional divisions to fade, fostering collaborative, holistic patient care but also exposing professionals to role dilution [15]. In this study, integrating these parallel frameworks provides the necessary conceptual architecture to analyze the empirical division of labor among DEs, shifting the focus from an isolated task list to a systemic analysis of operational realities.

## Methodology

### Study design and setting

**Fig. 1:**
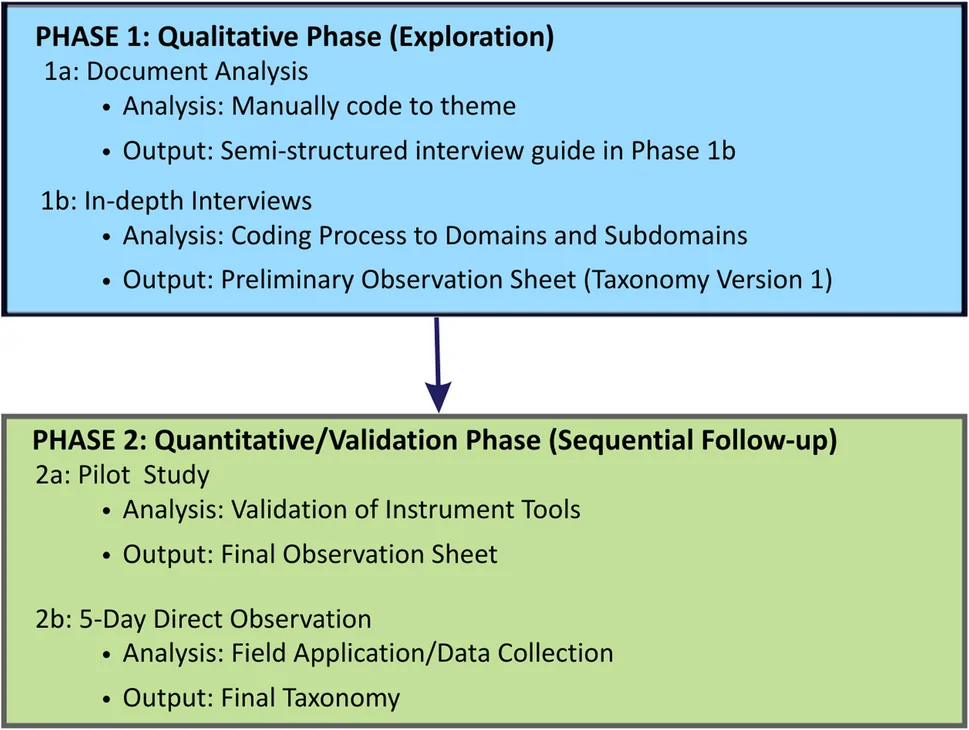
Flowchart of the sequential mixed-methods design: from qualitative exploration (Phase 1) to quantitative validation (Phase 2). This figure illustrates the sequential mixed-methods phases

This study utilized an exploratory sequential mixed-methods design to establish a comprehensive taxonomy of DEs working in MOH PHCs (Fig. 1). The study was conducted in two main phases:

- Phase 1 (Qualitative, Dec 2021 – Apr 2022): Document analysis and in-depth interviews (IDIs) to explore and define DE roles.
- Phase 2 (Quantitative, May – Sep 2022): Pilot study and direct observation to comprehensively capture and validate DE activities.

### Phase 1: Exploration of DE roles

- 1a. Document Analysis

A qualitative document analysis was conducted to establish baseline expectations for institutional roles and formal scopes of practice for DEs. Rather than an algorithmic or electronic keyword search of academic databases, a purposive expert-driven search process was employed. Relevant administrative, clinical, and policy documents were identified by mapping official publications from the MOH Malaysia and national professional societies (e.g., the Malaysian Diabetes Educators Society) against established international benchmark standards from the ADEA.

To ensure analytical rigor, strict inclusion and exclusion criteria were applied during this stage. Documents were included if they met the following parameters: (1) published by official governmental bodies or accredited national/international professional organizations; (2) directly outlined the clinical competencies, job descriptions, operational guidelines, or scopes of practice for DEs or allied primary care providers; and (3) were actively in use up to 2020 to capture the pre-study policy environment. Documents were excluded if they consisted of unverified internal clinic drafts, regional clinic-specific circulars without national endorsement, or clinical treatment protocols that omitted provider task specifications.

A final dataset of eight core documents (*n* = 8) met all eligibility criteria and were thoroughly analyzed. An overview and justification for the included document types are summarized in Table 1. Key information related to DE roles was manually extracted and entered into NVivo 12 for content analysis, where initial text codes were grouped into broader operational sub-domains to inform the subsequent semi-structured interview guides.

**Table 1:** Overview and justification of institutional documents reviewed (*n* = 8)

Document Name	Type	Year	Analytical Justification for Inclusion
Diabetes Education Manual 2020	Education Manual	2020	Establishes the standard baseline for national clinical competencies and education delivery.
Clinical Practice Guidelines Management of Type 2 Diabetes Mellitus, 6th Edition	Clinical Practice Guideline	2020	Outlines the formal interdisciplinary clinical pathway and role expectations within public healthcare.
Diabetes Education Manual 2016	Education Manual	2016	Provided a historical comparative baseline for the evolution of national educator competencies.
The 2017 Diabetes Educator and the Diabetes Self-Management Education National Practice Survey	National Practice Survey	2018	Used to benchmark the scope of professional practice against international, large-scale empirical metrics.
Role and Scope of Practice for Credentialled Diabetes Educators in Australia	Guideline	2015	Selected to benchmark domestic workflows against a highly mature international professional registration model.
ADEA National Standards of Practice for Credentialled Diabetes Educators	National Standards of Practice	2014	Evaluated structural quality indicators and task definitions of specialized educators globally.
*Senarai Tugas Nurse Diabetic Educator (Kesihatan Awam)*	Guideline	2020	Represents the official, binding public sector job description and administrative duties.
Diabetes educator role boundaries in Australia: A documentary analysis	Journal Article	2015	Provided empirical evidence regarding the negotiation of task boundaries in external primary care systems.

- 1b. In-Depth Interviews (IDIs)

Purposive sampling was used to select six expert participants involved in defining DE roles, career pathways, and decision-making. The inclusion criteria targeted individuals holding at least a senior management or specialist clinical status and over 5 years of administrative or operational oversight of the national primary care framework. The cohort included key stakeholders from the Nursing/Assistant Medical Officers Branches (*n* = 2), the Training Management Division (*n* = 1), and practicing senior DEs (*n* = 3) in the Klang Valley, the centralized administrative hub for the MOH in Malaysia. These participants were selected because their policy-making jurisdictions and administrative mandates dictate national practice standards for primary care frameworks across both urban and rural settings. Written informed consent was obtained.

The semi-structured interview guides used for this study were developed specifically for this research. The introductory script, consent forms (Format A), and participant information forms (Format B) were identical across all three guides. All the guides provided were in English. However, the IDI questions were tailored to the specific roles and perspectives of each participant group: implementers and leaders. The full guides are provided in the Supplementary Information (see Supplementary File 1: IDI Guide for Diabetes Educators; Supplementary File 2: IDI Guide for Leaders from Nursing/Assistant Medical Officer Branches; and Supplementary File 3: IDI Guide for Leaders from Training Management Division). The IDIs, lasting 30–45 min, were conducted in a private setting, audio-recorded, and transcribed verbatim. For this expert-elicitation phase, sample adequacy was determined by the depth of specialized institutional knowledge rather than participant volume. Because the six participants occupied the definitive leadership seats governing national DE training and deployment, this sample size provided comprehensive macro-level coverage of official role parameters.

Data saturation was determined when successive interviews with leaders across different administrative branches began to yield redundant operational sub-domains on standard operating procedures and job descriptions, indicating that the conceptual scope of official DE roles had been fully mapped.

### Content analysis

Content analysis for both the documents and IDIs was conducted using NVivo 12. An initial coding framework, based on sub-domains from the document analysis, was iteratively refined with new sub-domains emerging from the interview data. Meaningful text segments were categorized into preliminary sub-domains, which were then systematically organized into overarching domains reflecting DE roles and responsibilities. Discrepancies were resolved through consensus among research investigators. To ensure analytic oversight and mitigate individual interpretive bias, a researcher’s triangulation approach was employed, guided by established frameworks for consensus-building in team-based qualitative analysis [16, 17]. Specifically, transcription verification and initial thematic mapping were conducted in pairs, with all six research investigators participating. Following the initial paired coding, the qualitative verbatim data were systematically entered into NVivo 12 by NJN and PB. While formal inter-rater reliability statistics (e.g., Cohen’s kappa) were not calculated given the exploratory nature of the initial activity mapping, structural reliability was ensured through a recursive, consensus-based coding process. The research team held frequent peer-debriefing meetings to systematically cross-examine transcripts, preliminary codes, and observation logs. Any discrepancies in activity categorization, tier placement, or activity naming were debated extensively until a 100% collective agreement was reached. This rigorous approach ensured that the first version of the taxonomy accurately represents a collective, verified interpretation of the institutional data rather than a single researcher’s perspective. The final output served as the preliminary observation sheet for Phase 2.

### Phase 2: Validation and comprehensive data capture

- 2a. Pilot Study (Validation of Instrumental Tools)

Before the pilot study, five observers underwent training to standardize the use of the observation sheet, which adopted a time-motion study approach to capture the start and end times of each activity. These observers were research assistants holding diplomas or degrees in health-related fields, recruited specifically as dedicated data collectors for this project. Three PHCs in the Klang Valley were purposively selected for the pilot. To assess consistency, each observer was paired with a research investigator to independently and simultaneously observe a DE for a full working day. Feedback was collected and discussed by observers and research investigators to refine the content and structure of the second version of the taxonomy, which served as the final observation sheet for data collection.

- 2b. Direct Observation

The study population included all nurses and Assistant Medical Officers who held Post Basic Certificates or Advanced Diplomas in Diabetes Care and worked in the Outpatient Departments of MOH PHCs in a Malaysian state. This state was selected due to its high reported diabetes prevalence [18]. The sample size requirement was initially evaluated using the finite population correction mean formula [19] based on primary care time-motion parameters from existing literature. Due to the small, finite nature of this highly specialized workforce within the state (*N* = 60), the formulaic requirement (60–63 samples) either mirrored or exceeded the total absolute population ceiling. Consequently, traditional statistical sampling sub-bounds were bypassed in favor of a universal sampling approach to capture the empirical reality of the entire eligible state cohort. Strict exclusion criteria were subsequently applied, omitting individuals on long leave during data collection, those who had transferred out of the state, or those who did not provide written consent. Ultimately, a final active sample of 51 DEs across 43 PHCs was secured for the 5-day direct observation period, representing an exceptional 85.0% inclusion rate of the entire state’s population.

Prior to data collection, a meeting was held with top management and all participating DEs to explain the study objectives and secure written consent. Each trained observer monitored the same DE for five consecutive working days (Monday to Friday, excluding the lunch break). All DE activities, including clinical duties, administrative tasks, and other related activities (discussions, meetings, community outreach), were recorded on the final observation sheet. Data were entered into a Microsoft Excel sheet daily and regularly checked for accuracy. The comprehensive list of observed activities and tasks formed the basis for developing the final DE taxonomy.

### Synthesis and final taxonomy development

The final DE taxonomy was synthesized through an iterative integration of all data sources [20]. Phase 1 (document analysis and IDIs) provided the conceptual scope and preliminary activity list. This list was then subjected to empirical validation during the Phase 2 direct observations. Activities were refined, merged, or added based on their occurrence in the 43 observed PHCs. This transition from a theoretical framework to an observed reality ensured that the resulting 146 activities are both comprehensive and reflective of the actual daily workload of DEs in Malaysia.

The progressive expansion and categorization of the task taxonomy across the three development phases are systematically mapped in Table 2. As detailed in the table, the initial baseline clinical framework evolved through expert validation and real-world field observations, resulting in the final comprehensive distribution of operational domains and activities needed to accurately measure a DE’s actual clinical capacity.

**Table 2:** Methodological evolution and categorization of the task taxonomy

Phase/Version	Domain	Subdomain/ Activities	Chronological Justification for Category Expansion
Phase 1a (Initial Draft)	1. Diabetes Care	14 sub-domains	Focused strictly on the baseline clinical functions of diabetes management as outlined in standard literature and clinical practice guidelines.
Phase 1b (1st Taxonomy)	1. Diabetes Care 2. Non-Diabetes Care 3. Others	Diabetes Care: • 16 sub-domains • 45 activities Non-Diabetes Care: • 2 activities Others: • 1 activity	Expanded based on first-round expert feedback to acknowledge that DEs are occasionally drawn into cross-functional clinic tasks.
Phase 2b (Final Taxonomy)	1. Diabetes Care Non-Diabetes Care 2. Administrative 3. Supportive 4. Personal	Diabetes Care: • 16 sub-domains • 83 activities Non-Diabetes Care: • 4 sub-domains • 47 activities Administrative: • 10 activities Supportive: • 4 activities Personal: • 2 activities	**Final Verification Phase**: Micro-task observation revealed a major “hidden workload.” The clinical domain was split into Direct (face-to-face) and Indirect (registry/documentation) care. Purely operational domains (Administrative and Supportive) were introduced to fully account for actual daily clinical capacity.

To enhance the analytical utility of the taxonomy, activities were categorized into three groups based on the level of specialized expertise required: core specialized (high-impact activities requiring advanced certification in diabetes care, such as insulin stabilization or foot deformity screening); routine clinical (standard patient care tasks shared across the primary care team, including performing basic physical exams or vaccinations); and clerical/delegable (administrative coordination and manual documentation tasks that do not strictly require specialized expertise, including manual referral-letter writing and appointment logistics.). This classification serves as a conceptual tool for examining role dilution and systemic task shifting.

### Reflexivity and positionality

The research team acknowledged their positionality as practitioners and researchers within the MOH framework. To mitigate potential institutional bias or ‘top-down’ role definitions from Phase 1, the investigators employed extensive field-based validation in Phase 2. By observing 51 frontline DEs across 43 diverse clinics, the study prioritized ‘bottom-up’ empirical evidence over aspirational policy definitions, ensuring the taxonomy reflects actual practice rather than just official expectations.

## Results

**Fig. 2:**
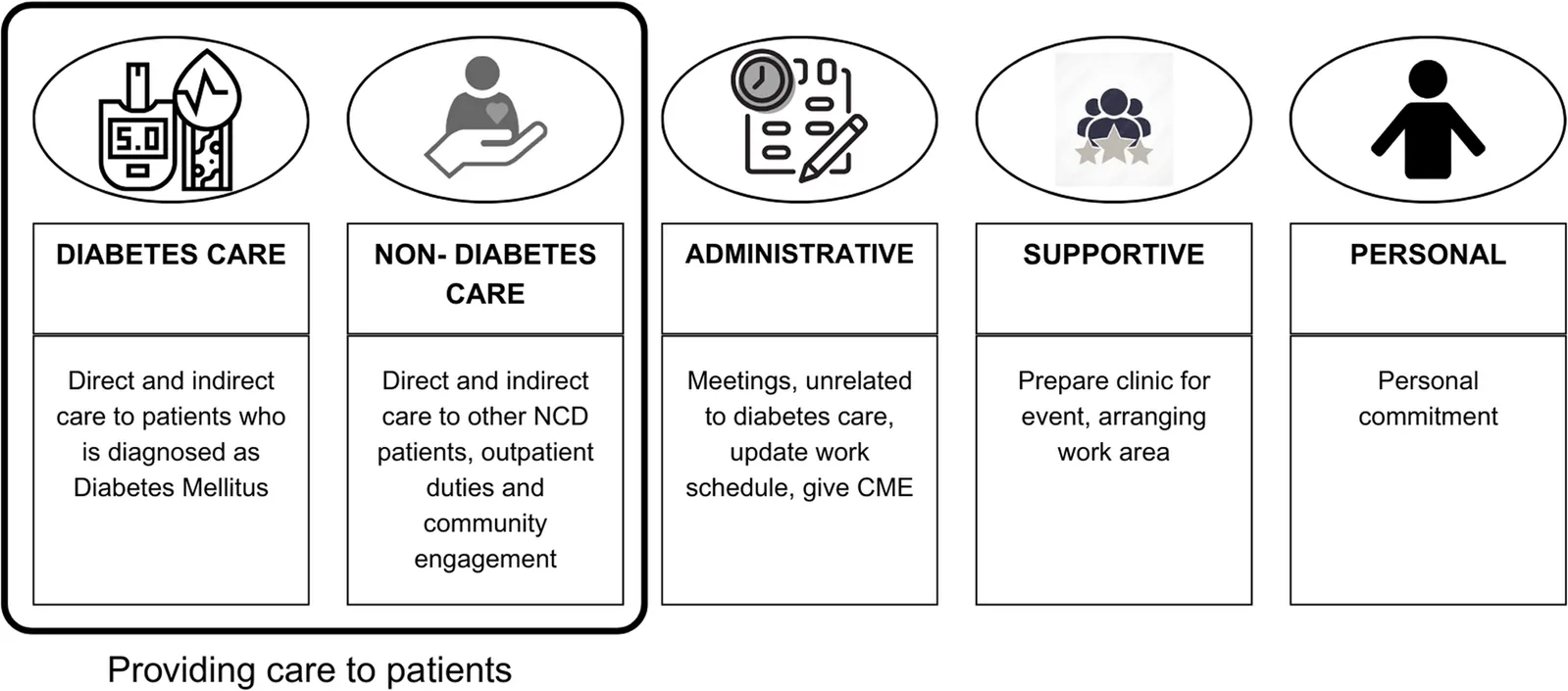
Five domains involving DEs’ activities in PHCs. This figure illustrates the conceptual grouping of DE tasks into diabetes care, non-diabetes care, administrative, supportive, and personal domains

**Fig. 3:**
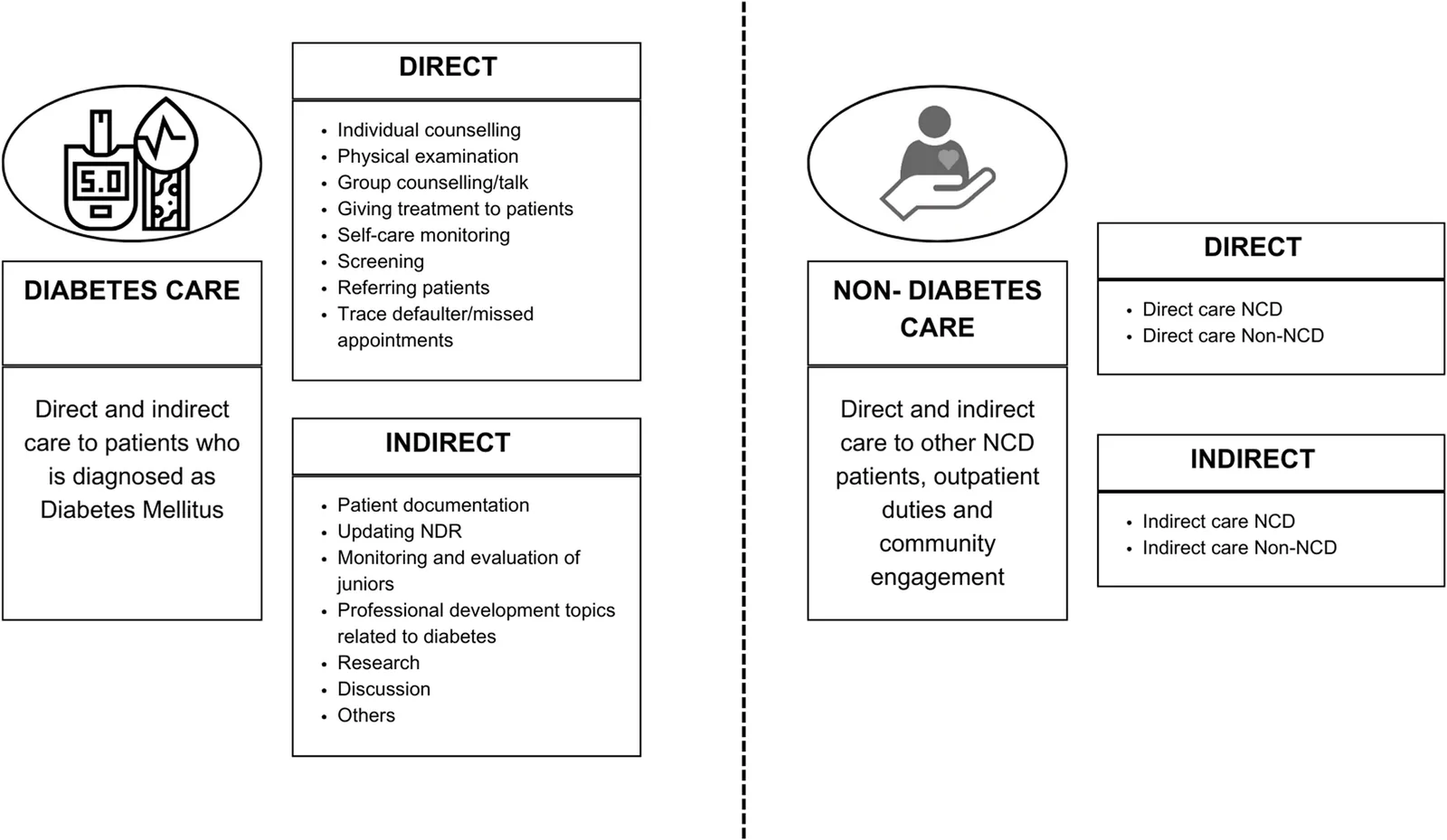
List of sub-domains for diabetes and non-diabetes care. This figure further analyzed into 15 sub-domains, including eight focused on direct patient care and seven on indirect patient care

Content analysis identified five distinct domains of activities performed by DEs in Malaysian primary health clinics (Fig. 2). Of these five domains, the two primary clinical domains, Diabetes Care and Non-Diabetes Care, were further delineated into direct and indirect care components across 15 and 4 sub-domains, respectively (Fig. 3). The comprehensive distribution of these 146 activities is summarized in Table 3, with the complete, highly detailed taxonomy provided in Additional File 1.

**Table 3:** List of activities within domains

Domains	Activities	Percentage (%)
Diabetes Care	83	56.85
Non-Diabetes Care	47	32.19
Administrative	10	6.85
Supportive	4	2.74
Personal	2	1.37
Total	146	100

A full detailed taxonomy is available in Additional File 1

Analysis revealed that while 56.85% of activities (83 tasks) were patient-centric, significant role dilution occurred, with 32.19% (47 tasks) dedicated entirely to non-diabetes care. The remaining activities were distributed across the administrative (6.85%), supportive (2.74%), and personal (1.37%) domains. Furthermore, when classified by professional boundaries, a massive 39.04% of all tasks were strictly clerical/delegable, significantly outstripping routine clinical (33.56%) and core specialized care (27.40%), underscoring a severe underlying administrative burden.

**Fig. 4:**
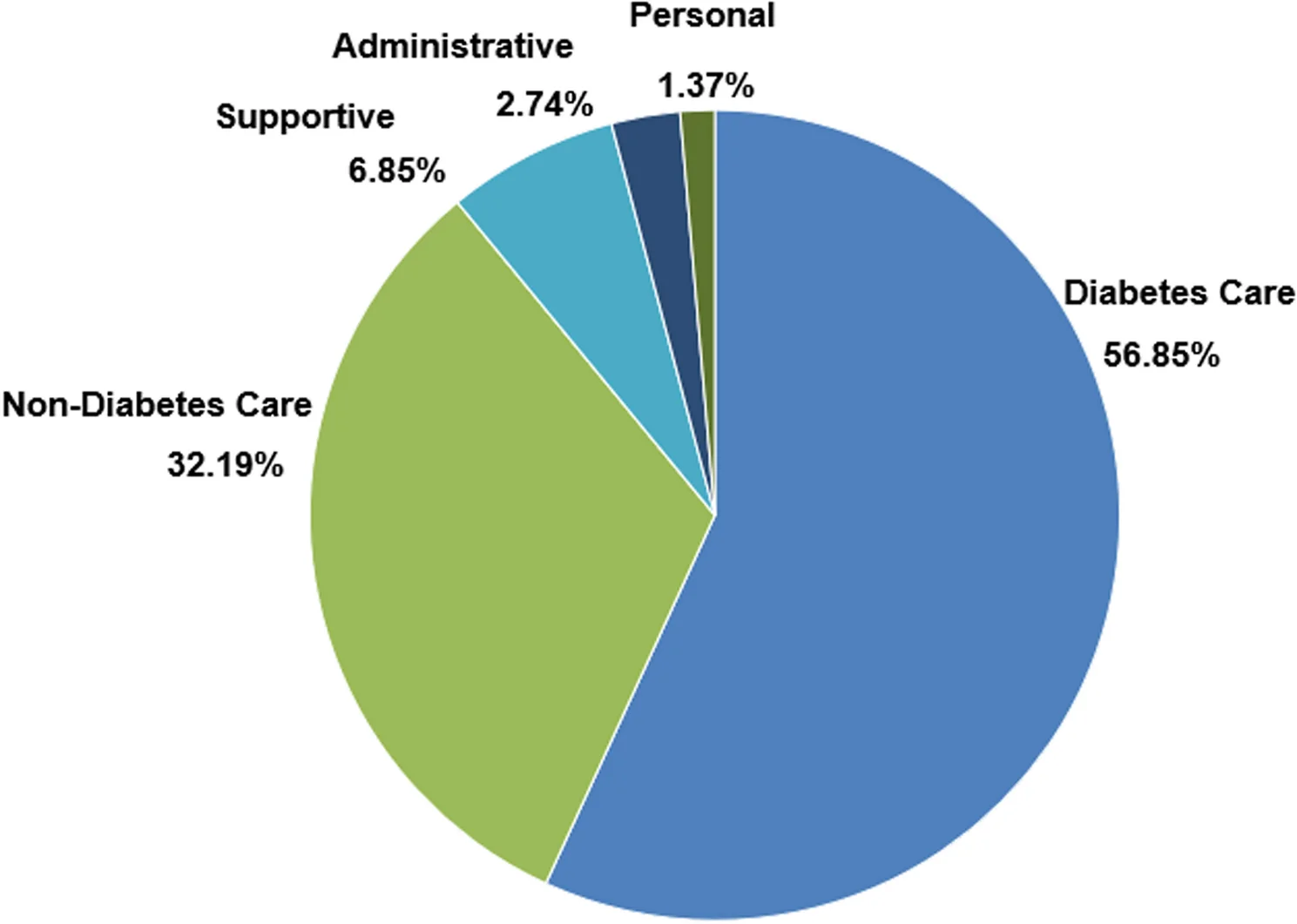
Percentage distribution of DEs’ activities by domains. Note: Percentages represent the diversity of activity types within the taxonomy and are not a measure of time-weighted workload intensity or total time spent

As in Fig. 4, the diabetes care domain accounted for 56.85% of all activities (83 activities), followed by the non-diabetes care domain at 32.19% (47 activities). The remaining activities were distributed across the administrative (6.85%, 10 activities), supportive (2.74%, 4 activities), and personal (1.37%, 2 activities) domains.

**Fig. 5:**
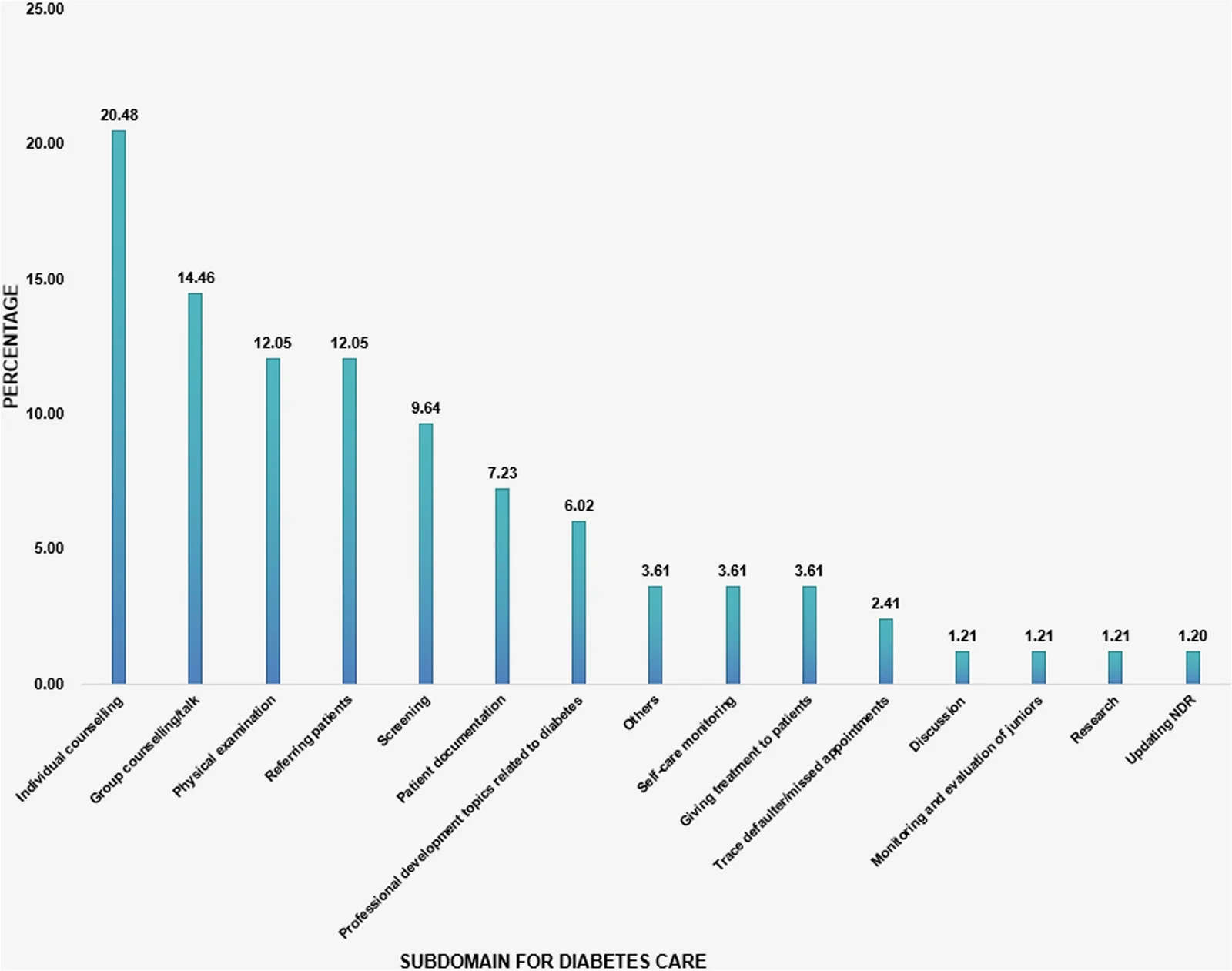
Percentage distribution of DEs’ activities by subdomain in diabetes care. Note: Percentages represent the diversity of activity types within the taxonomy and are not a measure of time-weighted workload intensity or total time spent

Within the diabetes care domain (Fig. 5), individual counseling accounted for the highest proportion of activity at 20.48%, followed by group counseling/talk (14.46%), physical examination, and patient referral at 12.05%, respectively.

**Fig. 6:**
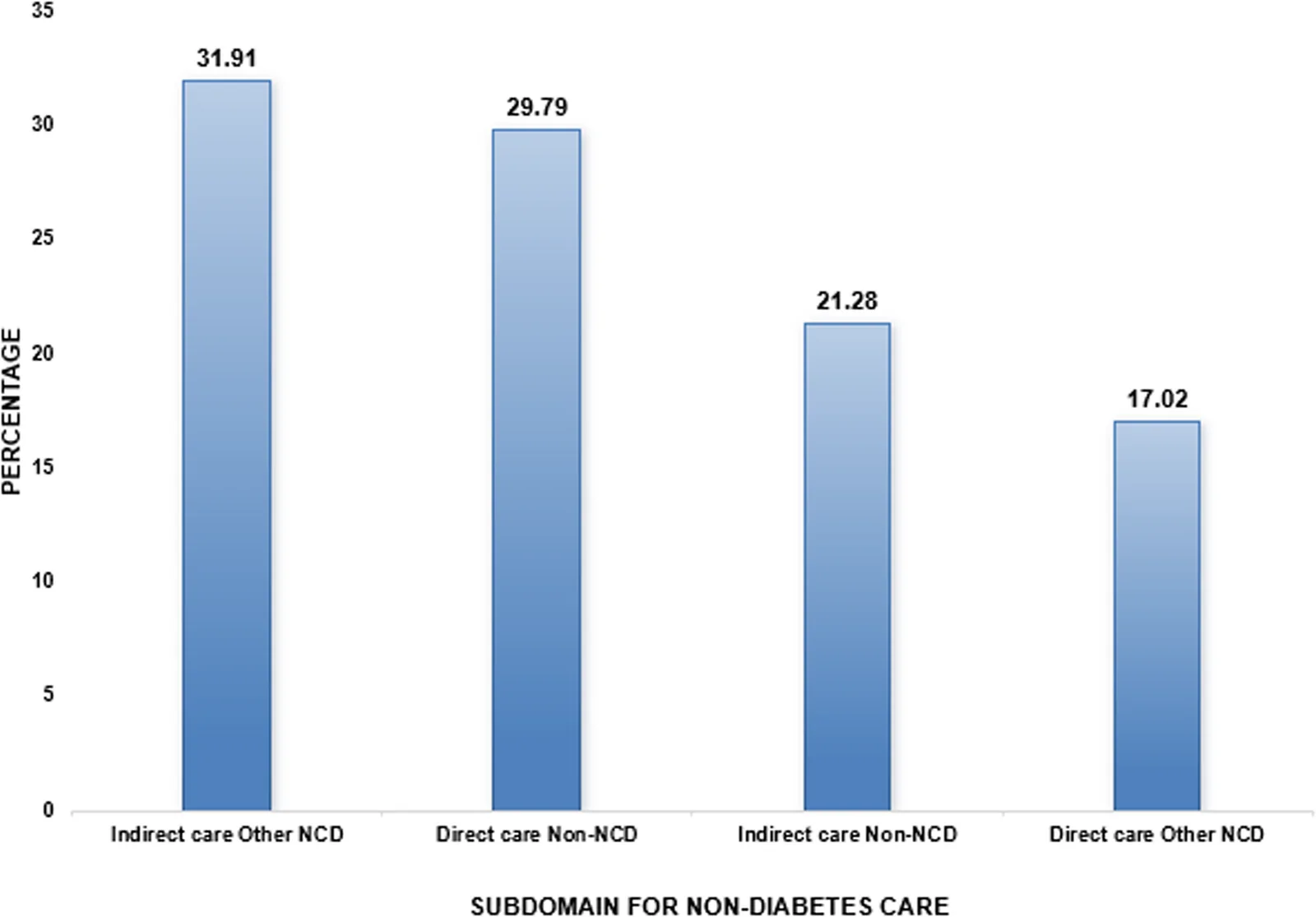
Percentage distribution of DEs’ activities by subdomain in non-diabetes care. Note: Percentages represent the diversity of activity types within the taxonomy and are not a measure of time-weighted workload intensity or total time spent

Within the non-diabetes care domain (Fig. 6), indirect care for other NCD patients was the most frequent activity at 31.91%, followed by direct care for non-NCD patients (29.79%).

## Discussion

The comprehensive taxonomy developed in this study provides a crucial, structured framework for defining the multifaceted roles and responsibilities of DEs within PHCs. By organizing the DE’s scope into five distinct domains and 146 specific activities, the taxonomy provides a practical tool to help DEs prioritize tasks and foster collaboration within multidisciplinary healthcare teams, thereby enhancing diabetes care management.

### Addressing role ambiguity and expertise utilization

While the initial role construction in Phase 1 relied on expert stakeholders, a practice that may carry a risk of ‘elite bias’ or aspirational role definitions, this study mitigated such bias through extensive Phase 2 validation. By observing 51 frontline DEs across 43 PHCs, the final taxonomy captures the day-to-day activities and operational realities of the role. This dual-phase approach ensures that the framework is not only theoretically sound but also grounded in the practicalities of the Malaysian primary care setting.

This framework directly addresses the long-standing challenges DEs face, particularly those who are nurses or Assistant Medical Officers burdened by general routine tasks and inefficient task division [5]. Literature highlights that DEs often experience a lack of clarity in their job descriptions, leading to varied interpretations of their roles and the underutilization of their specialized skills [21]. Other healthcare professionals may perceive DEs merely as general nurses, blurring their specialized responsibilities with general duties. The detailed clarification of roles provided by this taxonomy can reduce this role ambiguity and ensure the optimal deployment of the DEs’ specialized expertise [21].

Concerns regarding insufficient training and limited opportunities to deliver specialized services are also prevalent [21, 22]. Coupled with a shortage of trained DEs [21, 23], this creates critical gaps in patient supervision and self-management support, negatively impacting patient outcomes, especially since an estimated 60% to 70% of individuals with diabetes have not received adequate self-management training [24]. The findings underscore the continuous need for advanced qualifications and professional development to enhance DEs’ confidence and prepare them for more impactful, high-quality care delivery [25–27].

### Implications for care prioritization and management

The taxonomy developed in this study provides a detailed mapping of the breadth of DE activities. However, it is important to interpret these findings as reflections of activity frequency rather than time-weighted workload intensity. For instance, while patient-centric diabetes care accounted for 56.85% of identified activities, this reflects the high diversity and recurring nature of clinical tasks within this domain throughout the working day. This distinction is crucial for prioritization, as it highlights the breadth of clinical responsibilities DEs must manage, even if the individual duration of each task varies.

The economic benefits of dedicated diabetes education are well-documented, showing that patients receiving DE-led education incur lower overall healthcare costs [28, 29]. Given the time constraints of primary care providers, DEs are indispensable specialists in education and support [5, 28].

However, the reality in Malaysian PHCs is often fragmented care. While operating under a centralized MOH framework, care delivery remains non-standardized and tailored to individual clinic characteristics [5, 7]. This variability, combined with workforce shortages, results in programs that are multi-tasking and inefficient, leading to suboptimal outcomes. The taxonomy, by comprehensively mapping all activities, serves a crucial purpose. It helps Family Medicine Specialists (FMSs) and policymakers optimize workload distribution and ensure DEs can focus on their high-impact, specialized functions, which is crucial for achieving the enhanced outcomes associated with specialized diabetes teams [7].

Furthermore, the data shows DEs are crucial for diabetes data management, including inputting data into the National Diabetes Registry (NDR) [7]. The taxonomy highlights that while DEs are pivotal in this data-driven process, the system often relies on manual documentation, contrasting with the advanced technological integration seen in other developed healthcare systems [30]. The structured role definition can therefore inform strategies to improve data management practices in Malaysian PHCs [31]. By clearly delineating roles and facilitating prioritization, this taxonomy is a vital tool for streamlining care delivery and optimizing diabetes management across all PHCs.

### Critical interrogation of DE workload and role dilution

The findings of this study provide a granular activity-based taxonomy that explains the high administrative time allocation observed in recent Malaysian studies [32]. The high volume of activities classified as ‘clerical/delegable’ (39.04%) suggests that DEs are currently underutilized, indicating a potential for task-shifting to improve efficiency in primary care. This structural frequency provides the necessary context for interpreting the temporal burdens identified in similar primary care environments [32]. These findings reveal a stark contrast between the specialized intentions of the DE role and the operational realities of Malaysian PHCs. This reliance on manual registries and duplicative paperwork is not merely a matter of clinical governance; it is empirical evidence of systemic under-digitalization that forces highly trained specialists into clerical roles.

Furthermore, the observation that over one-third (32.19%) of activities involve non-diabetes care confirms a significant role of dilution. Viewed through the lens of professional boundaries, DEs are frequently pulled into general outpatient duties to compensate for staffing shortages, which compromises the delivery of intensive self-management education. This ‘task-shifting’ is compounded by ‘indirect care’ tasks classified as clerical/delegable, such as manual referral-letter writing and appointment logistics. While clinically necessary, the manual nature of these tasks represents a systemic inefficiency and a primary source of role dilution. Ultimately, by forcing specialists to prioritize administrative coordination, the current system inadvertently compromises their ability to deliver high-impact, ‘core specialized’ care. This empirical evidence underscores the urgent need for integrated digital health solutions to protect professional boundaries and optimize specialized nursing resources in Malaysian PHCs.

## Conclusions

DEs are pivotal to effective diabetes self-management and education, yet they frequently encounter significant challenges, including a lack of role clarity, insufficient training, and substantial administrative burdens. This study’s comprehensive taxonomy of DEs’ activities provides a structured framework that explicitly defines their diverse roles and responsibilities within PHCs. By detailing these activities, the taxonomy provides an essential empirical baseline to help healthcare administrators identify and structurally address inefficiencies stemming from undefined roles. This taxonomy provides concrete avenues for action, such as its direct integration into teaching syllabi and training curricula to standardize and enhance DEs education, serving as a robust foundation for strengthening policy and practice by enabling clearer task prioritization for DEs, facilitating enhanced team collaboration among healthcare professionals through clearly delineating DEs’ contributions, and ultimately leading to a more effective, coordinated, and sustainable approach to diabetes care management, fostering improvements in healthcare delivery and patient outcomes. While this study establishes a comprehensive mapping of the breadth and diversity of DE roles, the findings are based on activity frequency rather than time-weighted workload intensity. Future research incorporating time-motion analysis is recommended to refine task prioritization further and provide more granular data for workforce planning and resource allocation in primary health clinics.

### Limitations

This study has several limitations. First, a potential limitation is the small qualitative sample in Phase 1, which primarily involved stakeholders and senior educators. Although Phase 2 provided large-scale validation with frontline practitioners, the initial role construction might have benefited from including more junior DEs to capture a more contested or varied set of role perspectives. Second, the observation period was limited to five consecutive working days, excluding weekends. This duration may not have fully captured less frequent but potentially significant activities. Third, while the Hawthorne effect is a known limitation of direct observation, the study’s five-consecutive-day design allowed for a degree of habituation, in which participants became accustomed to the observer’s presence. Furthermore, the observation of 51 different DEs across 43 clinics provides a diverse cross-section of practice, minimizing the weight of any single participant’s behavioral change. Fourth, while manual observation provided a detailed view of activities, it inherently has limitations, such as unrecorded brief interactions or off-site activities performed by DEs that were not within the scope of direct observation. Finally, the study was conducted exclusively in PHCs within a single Malaysian state. Variability in managerial practices, patient demographics, and resource availability across different states or regions within Malaysia may affect the generalizability of the proposed taxonomy to the country as a whole.

## Supplementary Information

Supplementary Material 1.

Supplementary Material 2.

Supplementary Material 3.

Supplementary Material 4.

## Data Availability

The datasets generated and/or analyzed during the current study are not publicly available due to containing information that could compromise the privacy of research participants, but are available from the corresponding author on reasonable request.

## Abbreviations

CME: Continuous Medical Education
DE: Diabetes Educator
ECG: Electrocardiogram
FMSs: Family Medicine Specialists
HCP: Health Care Provider
HPT: Hypertension
IDI: In-depth Interview
MOH: Ministry of Health
NCD: Non-Communicable Disease
NCR: National Diabetes Registry
OPD: Outpatient Department
PHC: Primary Health Care

## References

[1] WHO. World Health Organization. https://www.who.int/health-topics/diabetes Accessed 14 November 2024. 2014.

[2] Institute for Public Health. National Health and Morbidity Survey (NHMS) 2023: Non-communicable Diseases and Healthcare Demand - Key Findings. Institute for Public Health, Ministry of Health Malaysia; 2024.

[3] Ismail R, Ismail NH, Mohd Tamil A, Ja’afar MH, Md Isa Z, Mat Nasir N, et al. Prevalence and factors associated with prediabetes and diabetes mellitus among adults: Baseline findings of PURE Malaysia cohort study. Clin Epidemiol Glob Health. 2023;21. 10.1016/j.cegh.2023.101279. PMC11256272 39035240

[4] Lim LL, Hussein Z, Noor NM, Abd Raof AS, Mustafa N, Long Bidin MB, et al. Real-world evaluation of care for type 2 diabetes in Malaysia: A cross-sectional analysis of the treatment adherence to guideline evaluation in type 2 diabetes (TARGET-T2D) study. PLoS ONE. 2024;19(1 January). 10.1371/journal.pone.0296298 . PubMed PMID: 38166049. PMC10760741 38166049

[5] Mustapha FI, Aagaard-Hansen J, Lim SC, Nasir NH, Aris T, Bjerre-Christensen U. Variations in the Delivery of Primary Diabetes Care in Malaysia: Lessons to Be Learnt and Potential for Improvement. Health Serv Res Manag Epidemiol. 2020;7. 10.1177/2333392820918744. PMC7160766 32313820

[6] Ministry of Health Malaysia. Clinical practice guidelines: management of type 2 diabetes mellitus (6th edition). Putrajaya: Ministry of Health Malaysia, Academy of Medicine Malaysia, Malaysian Endocrine & Metabolic Society, Diabetes Malaysia and Family Medicine Specialists Association of Malaysia. 2020.

[7] Hussein Z, Taher SW, Gilcharan Singh HK, Chee Siew Swee W. Diabetes Care in Malaysia: Problems, New Models, and Solutions. Annals of Global Health. Elsevier USA; 2015. pp. 851–62. 10.1016/j.aogh. 2015.12.016 PubMed PMID: 27108152. 27108152

[8] Grohmann B, Espin S, Gucciardi E. Patients’ experiences of diabetes education teams integrated into primary care. Can Fam Physician. 2017;63(2):e128–36. PMC5395411 28209705

[9] Australian Diabetes Educators Association. Role and scope of practice for credentialled diabetes educators in Australia. Canberra: ADEA; 2015.

[10] Davis J, Fischl AH, Beck J, Browning L, Carter A, Condon JE, et al. 2022 National Standards for Diabetes Self-Management Education and Support. Diabetes Care. 2022;45(2):484–94. 10.2337/dc21-2396 . PubMed PMID: 35231087. 35050365

[11] Disease Control Division, Ministry of Health Malaysia. Senarai tugas nurse diabetic educator (kesihatan awam). Putrajaya: Ministry of Health Malaysia; 2020. [Unpublished document].

[12] Gucciardi E, Xu C, Vitale M, Lou W, Horodezny S, Dorado L, et al. Evaluating the impact of onsite diabetes education teams in primary care on clinical outcomes. BMC Fam Pract. 2020;21(1). 10.1186/s12875-020-01111-2. PubMed PMID: 32126965. PMC7055112 32126965

[13] Malhotra K, Ali A, Soran V, Ogiliev T, Zhou D, Melson E, et al. Levelling the learning ground for healthcare professionals across the world through SIMBA: a mixed-methods study. BMJ Open. 2023;13(7). 10.1136/bmjopen-2022-069109. PubMed PMID: 37429686. PMC10335543 37429686

[14] Orkin AM, Rao S, Venugopal J, Kithulegoda N, Wegier P, Ritchie SD, et al. Conceptual framework for task shifting and task sharing: an international Delphi study. Hum Resour Health. 2021;19(1). 10.1186/s12960-021-00605-z. PubMed PMID: 33941191. PMC8091141 33941191

[15] Eliassen M, Moholt JM. Boundary work in task-shifting practices–a qualitative study of reablement teams. Physiother Theory Pract. 2023;39(10):2106–19. 10.1080/09593985.2022.2064380 . PubMed PMID: 35430950. 35430950

[16] Patton MQ. Enhancing the quality and credibility of qualitative analysis. Health Serv Res. 1999;34(5 Pt 2):1189–208. PMC1089059 10591279

[17] Hill CE, Knox S, Thompson BJ, Nutt Williams E, Hess SA, Nutt E. Consensual Qualitative Research: An Update Consensual Qualitative Research: An Update Recommended Citation Recommended Citation [Internet]. Available from: https://epublications.marquette.edu/edu_fac/18

[18] Institute for Public Health. National Health and Morbidity Survey (NHMS) 2019: Vol. I: NCDs – Non-Communicable Diseases: Risk Factors and other Health Problems. Shah Alam: Institute for Public Health, Ministry of Health Malaysia; 2020.

[19] Lwanga SK, Lemeshow S. Sample size determination in health studies: a practical manual. Geneva: World Health Organisation; 1991.

[20] Institute for Health Management. The role of diabetes educators and time spent for diabetes care in primary health clinics, Negeri Sembilan. Shah Alam: Institute for Health Management, National Institutes of Health, Ministry of Health Malaysia; 2023.

[21] Saidi S, Milnes LJ, Griffiths J. Are we doing it right? Self-care support for patients with type 2 diabetes in urban areas in Malaysia. Enferm Clin. 2019;29:691–7. 10.1016/j.enfcli.2019.04.106.

[22] Davis ED. Role of the diabetes nurse educator in improving patient education. Diabetes Educ. 1990;16(1):36–8. doi:doi:10.1177/014572179001600109 PubMed PMID: 2311497. 10.1177/014572179001600109 2311497

[23] Ramli A, Taher S. Managing chronic disease in the malaysian primary health care - a need for change. Malaysian Family Physician [Internet]. 2008. Available from: http://www.ejournal.afpm.org.my/PMC4267020 25606105

[24] Austin MM. Diabetes Educators: Partners in Diabetes Care and Management. Endocr Pract. 2006;12(Suppl 1):138–41. 10.4158/EP.12.S1.138 . PubMed PMID: 16627398. 16627398

[25] Ross K, Camara K, Waldron S, Kime N. The evolving role of the diabetes educator. Diabetes Care Child Young People. 2016;5(1):23–8.

[26] Hodgkins A. Diabetes education in primary care: target sessions. J Diabetes Nurs. 2009;13(5):186–91.

[27] Jacqueline D, Dudley. The diabetes educator’s role in teaching the diabetes patient. Diabetes Care. 1980;3(1):127–33. 10.2337/diacare.3.1.127. 7408603

[28] Burke SD, Sherr D, Lipman RD. Partnering with diabetes educators to improve patient outcomes. Diabetes, Metabolic Syndrome and Obesity. Dove Medical Press Ltd; 2014. pp. 45–53. 10.2147/DMSO.S40036. PMC3926770 24550679

[29] Duncan I, Birkmeyer C, Coughlin S, Li Q, Sherr D. Assessing the value of diabetes education. Diabetes Educ. 2009;35(5):752–60. 10.1177/0145721709343609 . PubMed PMID: 19783766. 19783766

[30] James S, Perry L, Gallagher R, Lowe J. Diabetes Educators Perceived Experiences, Supports and Barriers to Use of Common Diabetes-Related Technologies. J Diabetes Sci Technol. 2016;10(5):1115–21. 10.1177/1932296816660326. PMC5032969 27417141

[31] Wetterneck TB, Lapin JA, Krueger DJ, Holman GT, Beasley JW, Karsh BT. Development of a primary care physician task list to evaluate clinic visit workflow. BMJ Qual Saf. 2012;21(1):47–53. 10.1136/bmjqs-2011-000067 . PubMed PMID: 21896667. PMC3568931 21896667

[32] Noris NJ, Bakit P, Saimy IS, Libasin Z, Khairul Anuar IL, Wan Alias WNA, et al. Quantifying and understanding the roles of diabetes educators in Malaysian primary health clinics: a mixed-methods study. BMC Health Serv Res. 2026;26(1). 10.1186/s12913-026-14144-7. PMC12903666 41634715

