# Supplementary material for: Professional boundary blurring and role dilution: a taxonomy of diabetes educator activities in Malaysian primary care
Source: BMC Prim Care. 2026 Jul 29;27:386. doi: 10.1186/s12875-026-03489-x (PMC13711968; doi:10.1186/s12875-026-03489-x)
Supplement: Supplementary file 2 [file 12875_2026_3489_MOESM3_ESM.pdf]


For office use only

Understanding the Role of Diabetes Educators and Measuring Their Time Spent for Diabetes Care in Ministry of Health Primary Health Clinics.

NMRR: NMRR-21-1045-59255

MINISTRY OF HEALTH MALAYSIA

A GUIDE FOR THE CONDUCT OF A

"IN-DEPTH INTERVIEW (IDI)"

**RESPONDENT**	
**INTERVIEWER**	
**NOTE-TAKER**	

Essential Tools:

1. Participant information sheet
2. Consent forms
3. Evaluation sheets, one for each participant
4. Pads & Pencils for participant
5. IDI Guide for interviewer
6. 1 recording device
7. Batteries for recording device
8. Extra tapes for recording device
9. Notebook for note-taking
10. Refreshments

IDI GUIDE

I Preparation before IDI sessions

- Preparing question guide for IDI
- Inform participants when to conduct IDI
- Identify the place and date for IDI
- Prepared digital recorder, notebooks for note takers, snacks and drink for participants

II Before the discussion

- Arrive early, Prepare the snacks and drinks
- Arrange the seating
- Ensure privacy
- Prepare materials (paper, pen, digital recorder, battery)
- Check digital recorder to ensure good functioning

III During the discussion

- Self-introduction
- Introduce "Note Taker"
- Explain the purpose of this study
- Fill up the written consent form (refer Format A)
- Take personal information (refer Format B)
- Topic for discussion
  Roles of Diabetes Educators in primary health clinics
- Use of data: As an input to make a recommendation to the top management/policy makers
- Duration: 30 minutes to 1 hour
- Ethics: guarantee of anonymity, confidentiality
- Asking permission to tape recorded the session
- Rules of the session:
- There is no RIGHT or WRONG answer
- Do not be afraid to share a view.

IV Closing (End of Discussion)

- Summarize key ideas and counter-checking with participant
- Reiterate confidentially
- Thank respondent

V After the IDI sessions

- Review and complete notes
- Discuss IDI atmosphere (e.g: smooth, team coordination, etc based on observation)
- Check the recording result

VI Analysis of IDI

DID CHECKLIST FOR NOTE TAKER

BEFORE the session

No.	Activities	YES	NO
1	Familiarize yourself with the objectives of this IDI		
2	Familiarize yourself with the process, questions and probe of this IDI		
3	Assist in organizing the logistic of the session		
4	Arrive early in the IDI room		
5	Ensure your IDI guide is available		
6	Test the digital recorder		
7	Test the tape voice capture		
9	Ensure all the digital recorder are labelled correctly		
10	Ensure enough number of reporting form (paper or notebook, pen available)		
11	Familiarize yourself with all the formats (reporting formats)		

DURING the session

No.	Activities	YES	NO
1	Build rapport with the participant		
2	Introduce yourself		
3	Counter-check digital recorder labelling		
4	Ensure digital recorder is switched on		
6	Ensure that IDI reporting form is available		
8	Take note of everything discussed during the meeting		
9	Ensure the digital recorder is working		
10	Ensure that all recorders are labelled correctly		
11	Counter-check all questions in IDI guide have been addressed		
12	Take note all non-verbal communication		
13	Summarise the whole environment by observing the session		

The main roles of the IDI “Note Taker” during the discussion:

- As an observer to the interview:
  - Responsible to take note of the discussion atmosphere
  - Be an active observer
- As a note taker of the interview:
  - Able to follow the discussion closely and record notes legibly
  - As far as possible, the exact words and phrases used by the participant are to be noted
  - Make sure adequate supply of writing material is available
- Manage the digital recorder:
  - This is the only back up for note taking
  - Make sure the tape is functioning properly
  - Trial run before the session
- Assist the interviewer:
  - Assist in organizing the meeting
  - To clarify statements
  - To suggest new relevant questions (if any)
  - To discreetly remind the interviewer on checklist of questions
  - To discreetly remind the interviewer on the time
  - To counter check coverage of questions/issues discussed
  - To remind the facilitator on refreshments

- Write up of report:
    - After review of notes with facilitator
    - To summarize discussion in written report to the facilitator
    - Label the digital recorder used in each discussion
    - Hand over notes and digital recorders/tapes to the facilitator together with written report

FORMAT A

PARTICIPANT INFORMATION SHEET AND INFORMED CONSENT FORM

Title of study: Understanding the Role of Diabetes Educators and Measuring Their Time Spent for Diabetes Care in Ministry of Health Primary Health Clinics.

NMRR: NMRR-21-1045-59255

1. **Name of investigator and institution: Ministry of Health**

   Principal Investigator: [Principal Investigator Name]

   Co-investigators:

   - [Co-investigator Name]
   - [Co-investigator Name]
   - [Co-investigator Name]
   - [Co-investigator Name]
   - [Co-investigator Name]

2. **Name of sponsor: Ministry of Health Malaysia Research Grant**

3. **Introduction:**

It is important that you understand why the research is being done and what it will involve. Please take your time to read through and consider this information carefully before you decide if you are willing to participate. Ask the study staff if anything is unclear or if you would like more information. After you are properly satisfied that you understand this study, and that you wish to participate, you must sign this informed consent form.

Your participation in this study is voluntary. You do not have to be in this study if you do not want to. You may also refuse to answer any questions you do not want to answer. If you volunteer to be in this study, you may withdraw from it at any time. If you withdraw, any data collected from you up to your withdrawal will still be used for the study. Your refusal to participate or withdrawal will not affect any medical or health benefits to which you are otherwise entitled.

This study has been approved by the Medical Research and Ethics Committee, Ministry of Health Malaysia.

4. What is the purpose of the study?

The purpose of this study is to understand the role and tasks expected of a trained Diabetes Educator (DE) in primary health clinic, and to evaluate the utilisation of time by DE for diabetes care in primary health clinics.

This research is necessary to improve the understanding on the role of DEs in the health clinics, Ministry of Health Malaysia (MOH). This research will be conducted for duration of 12 months (16 December 2021 till 31 December 2022). This research will be divided into two phases, Phase 1 (16 December 2021 till 28 February 2022) and Phase 2 (1 March 2022 till 30 November 2022). The expected number of participants is eight in Phase 1 and 53 individuals in Phase 2.

Diabetes educators (DE) have been defined as healthcare professionals whose practice focuses on educating individuals with and at risk for diabetes and related conditions to become effective self-managers. DEs play an important role in equipping patients with the knowledge and confidence to achieve self-care goals for metabolic control. With the increasing cases of DM in the country, the role of DE is very important in managing DM alongside the other healthcare professionals such as doctors, pharmacists. Diabetes educators are increasingly being recognised of its importance and accredited in many parts of the world.

In Malaysia, DEs are trained for six months in diabetes management at Institut Latihan Kemahiran Kementerian Kesihatan Malaysia (ILKKM). After the completion of the course, they are either being placed in hospitals or health clinics. Since most of the diabetes patients were seen in primary health clinics, 66.35% of DEs are placed in primary care. After their placement, the DEs are expected to perform duties related to diabetes care but sometimes they may not be able to do expected duties optimally due to multiple reasons that need to be explored.

5. Procedure and Responsibilities when taking part in this study?

Participation in this project will involve attending an interview that will discuss on the roles and functions of Diabetes Educators (DE) in MOH primary health clinics (PHCs). The participant is key personnel to the development of DE in MOH and important in governing the DEs who is comprised of staff nurses and assistant medical officers. The participant is also those important in governing and supervising the management and running of diabetes activities in PHCs. The participant of the interview will also include a representative from group of DE from several PHCs. We, the researchers will be present to conduct the interviews. The interview sessions will be conducted in Bahasa Malaysia and English. Duration of each interview is estimated to be 30-40minutes. There will eight participants that will be interviewed in this phase.

The interview sessions will be digital-recorded and transcribed for analysis. MOH does not permit us to supply copies of data to participants. The recordings will be used for analysis purposes only and will not be copied/sent to any other individual or used for any other purposes. After analysis, the recordings will be disposed of securely.

It is important that you answer all of the questions asked by the study staff honestly and completely.

6. Potential Risks

The risk is minimal. You are free to decline to answer any of the questions that you feel uncomfortable with. Your participation may be terminated prematurely if you withdraw your consent or if the investigators are unable to sustain or further manage the study.

7. Benefits of being in this study

There may or may not be any benefits to you. Information obtained from this study will help devising possible strategies to improve DE services, possible measures to be implemented in future MOH policy as recommended to the stakeholders.

8. Funding of the Research

This study is sponsored by Ministry of Health Malaysia Research Grant. You will not be paid for participating in this study.

9. Privacy and Confidentiality

All your information obtained in this study will be kept and handled in a confidential manner, in accordance with applicable laws and/or regulations. The analysis will be done in a way that prevents the identification of individuals. Only study team members, qualified monitors and auditors, and governmental or regulatory authorities may inspect the study data, where appropriate and necessary.

Participants will not be given access to study data unless data clarification is needed. However, participants may write to the investigators to request access to study findings upon completion of study. Research output will be presented anonymously without unique identifications of respondents.

Publications of study findings will be shared with participants either on an individual or group basis. When publishing or presenting the study results, your identity will not be revealed without your expressed consent.

10. Questions/Queries

If you have any questions about the study or you want information about this study, please contact the study investigator, [Principal Investigator Name] at [Primary Investigator Contact Details].

If you have any questions about your rights as a participant in this study, please contact: The Secretary, Medical Research & Ethics Committee, Ministry of Health Malaysia, [MREC Contact Details].

INFORMED CONSENT FORM

Title of Study: Understanding the Role of Diabetes Educators and Measuring Their Time Spent for Diabetes Care in Ministry of Health Primary Health Clinics.

By signing below I confirm the following:

- I have been given oral and written information for the above study and have read and understood the information given.
- I have had sufficient time to consider participation in the study and have had the opportunity to ask questions and all my questions have been answered satisfactorily.
- I understand that my participation is voluntary and I can at any time free withdraw from the study without giving a reason. I understand the risks and benefits, and I freely give my informed consent to participate under the conditions stated. I understand that I must follow the study investigator's instructions related to my participation in the study.
- I understand that study staff, qualified monitors and auditors, the sponsor or its affiliates, and governmental or regulatory authorities, have direct access to my information in order to make sure that the study is conducted correctly, and the data are recorded correctly. All personal details will be treated as STRICTLY CONFIDENTIAL
- I will receive a copy of this subject information/informed consent form signed and dated to bring home

Having considered the above, I hereby: (Please tick your choice)

☐ Agree to voluntarily take part in this study as a participant.

☐ Do not agree to voluntarily take part in this study as a participant.

Participant

Signature: **I/C Number:**

Name: **Date:**

Investigator conducting informed consent

Signature: **I/C Number:**

Name : **Date:**

FACILITATOR GUIDE (IDI)

Topics	Suggested Script	Instruction to Moderators
Welcome	"Good morning. Thank you for agreeing to participate in this interview<br><br>I am ______________________ , your interviewer from __________________. With me is _________________, our note-taker	*[Know the names of your note-taker before start of the interview]*<br><br>*[look towards note-taker].*
Purpose	The purpose of our interview today is to gather your views on the role of Diabetes Educator. We also like to get your inputs and suggestion regarding DE.<br><br>The information you provide is confidential and will only be used for this project.	
Consent	We would like to reassure you that the information that you provide is totally **confidential** and will not be disclosed to irrelevant parties. Your name, address, and other personal information will be removed, and only a dummy code will be used to connect your name and your responses. There is no right or wrong answers.<br><br>Your participation in this discussion is **voluntary.** You are free to refuse to answer any question.	
Caution about the recording	This interview will be recorded to enable us to transcribe and analyse it systematically.	
Ground rules	Before we begin, we would like to seek your cooperation in adhering to a few ground rules so that the interview can be conducted smoothly. Also it would make the work of transcribing much easier.<br><br>Firstly, please keep to the topic of the discussion.<br>Please stay calm and focused throughout the interview<br>Since this is an important interview, we would like to keep minimal external disturbance including cell-phone.<br><br>May I now invite you to introduce yourself and your organization.	

Question Guide For Nursing and Assistant Medical Officer Branches

1	Perception of the role of Diabetes Educator	1. What do you understand about the role of a DE working at a Health Clinic?	— For example, health education and counseling, what is their role in this aspect.<br>— What scope do they need to cover when providing education to patients, are there guidelines or checklists?<br>— Can you tell me a little about the guidelines? (If he mentioned there are guidelines)<br>— As I was informed, there is an annual screening for DM patients, which includes foot care, fundus exam and so on.<br>— Who should do the screening e.g. foot care, fundus - is it the responsibility of the DE or can other staff do this task?<br>— What about the National Diabetes Registry (NDR) data?<br>— Who should do the data entry and update of the NDR?<br>— What is your opinion about GDM patients in KK. Do they all need to be seen by the DE?
		2. From what you mentioned earlier, what role do you think is the most important regarding DE?	— Can you explain why you think that is the most important?

2	Supervising and monitoring of DE	3. What about the supervision of DE?<br><br>4. Regarding the Key Performance Indicators (KPIs) of a DE in a health clinic. Are there specific KPIs for their roles as a DE?	– Who is responsible for monitoring them?<br><br>– Can you please explain further how and who created this KPIs, and what is it based on?<br><br>– Does the DE need to send any reports to the Nursing and Assistant Medical Officer Branches regarding their roles and achievements as a DE?<br><br>– When we interviewed the DE, they mentioned sending a report. What is contained in this report and who monitors it?
3	Expectation	5. What are your expectations or what do you expect from DE?	– To what extent do you hope they can do their job as a DE?<br>– Is every day focused and doing diabetes care work or what?<br><br>– Do you feel that their work as a DE so far is sufficient or do you feel that they can do more?

1. Wrapping up

Comment on the interview and participation.

2. Closing

3. Before closing, you may want to ask:

For all the things that we have discussed and before we end this session, do you have any other things to add? Thanking the participant - express your appreciation on the input and time. State that it will certainly help us in making changes. Reaffirm on the confidentiality of the information gathered.

"That concludes our IDI. Thank you so much for coming and sharing your thoughts and opinions with us. We have a short evaluation form that we would like you to fill out if you time. If you have additional information that you did not get to say in the focus group, please feel free to write it on Format B form."

FORMAT B

PARTICIPANT INFORMATION

Place : ___________________________ **Date of IDI :** __________________________

Interviewer : ______________________ **Start Time :** ____________________

Note taker : ______________________ **End Time :** ____________________

IDI Session : Understanding the Role of Diabetes Educators and Measuring Their Time Spent for Diabetes Care in Ministry of Health Primary Health Clinics.

Name	Age	Position	Year of 1st Service as DE	Work Place

Comment :

